# The role of intracerebral dopamine D1 and D2 receptors in sleep‐wake cycles and general anesthesia

**DOI:** 10.1002/ibra.12024

**Published:** 2022-02-21

**Authors:** Jie Zhang, Jia Li, Cheng‐Xi Liu, Huan Gui, Cheng‐Dong Yuan, Yi Zhang

**Affiliations:** ^1^ The Second Affiliated Hospital of Zunyi Medical University Zunyi China; ^2^ Guizhou Key Laboratory of Anesthesia and Organ Protection Zunyi Medical University Zunyi China; ^3^ School of Anesthesiology Zunyi Medical University Zunyi China

**Keywords:** dopamine, D1 receptor, D2 receptor, emergence, general anesthesia, sleep‐wake

## Abstract

Dopamine (DA), a monoamine neurotransmitter, is synthesized and released mainly by neurons in the ventral tegmental area and the substantia nigra (SN) pars compacta of the midbrain. DA and its receptors are essential for the regulation of arousal, movement, cognition, reward, and other neurobiological behaviors. Arousal, locomotion, cognition, reward, and other neurobiological functions are all regulated by dopamine and its receptors. Dopamine receptors can be divided into D1‐like receptors (including D1 and D5) or D2‐like receptors (containing D2, D3, and D4), with D1 and D2 receptors (D1Rs, and D2Rs) being the most important. Currently, studies indicated that D1Rs and D2Rs are tightly involved with the process of sleep‐wake and general anesthesia, but the specific mechanism remains unclear. In this review, we compiled the most recent findings, mainly focusing on the structure, distribution, and signal pathway of D1Rs and D2Rs in the central nervous system, as well as the involvement of D1Rs and D2Rs in sleep‐wake and general anesthesia. Thus, the investigations of the D1Rs and D2Rs will benefit not only better knowledge for how sleep‐wake control works but also the mechanism of general anesthesia.

## Graphical abstract

The distribution of dopamine D1 and D2 receptors in the central nervous system is shown here. Activation of D1R in NAc, DRN, and NB promotes awakening from natural sleep and general anesthesia. D2R acts in a brain area‐dependent manner, with D2R in NAc playing a sleep‐promoting role and D2R in DRN and ventral tegmental area a awakening‐promoting role, and the function of both receptors in other brain areas remains to be further investigated.

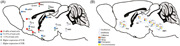

## 1 INTRODUCTION

General anesthesia is considered as an anesthetics‐induced state that can be reversed, causing unconsciousness, anterograde amnesia, immobility, and analgesia. Traditionally, the emergence from general anesthesia is a mirror image passive process of the induction period of general anesthesia. Many serious complications are related to general anesthesia, such as delayed recovery,^1^ restlessness,^2^ and delirium.^3^ Consequently, there has been a renewed interest in how to accelerate the recovery of general anesthesia, prevent convalescent complications, and improve the quality of postoperative resuscitation. Nevertheless, from the molecular level to brain circuit mechanisms, we still do not have a complete picture of how general anesthesia works.

D1Rs and D2Rs have been linked to changes in consciousness during sleep‐wake cycles and general anesthesia in various research in recent years.^4^, ^5^, ^6^ Therefore, a narrative review of the possible D1Rs‐ and D2Rs‐related neural mechanism of consciousness modulation during sleep‐wake and general anesthesia is presented, along with a discussion of their related structure, distribution, and signal transduction pathways.

## 2 STRUCTURE OF D1RS AND D2RS

DA receptor is a receptor family composed of seven transmembrane domains that can be coupled with guanylate‐binding protein. Based on the structural, pharmacological, and biochemical properties of DA receptors, they were divided into five subtypes, namely Dl, D2, D3, D4, and D5.^7^, ^8^, ^9^, ^10^ In the central nervous system (CNS), DA receptors are mainly D1Rs and D2Rs. The molecular weight of D1Rs and D2Rs is about 50 kDa. The D1R gene is located on human chromosome 5 q35, which has only exons, and the mRNA size of D1R is 3.8 kb, encoding 446 amino acids. The D2R gene, containing intron and exon, is found on chromosome 11 q22‐23 and its mRNA size is 2.5 kb, which encodes 415‐444 (mouse) and 414‐443 (human) amino acids. Selective splicing of D2 leads to the production of two major D2 dopamine receptor variants, containing D2‐short (D2S) and D2‐long (D2L). D2LR has 29 more amino acid sequences compared with D2SR in the third intracellular loop.^11^ The amino acid chain is N‐terminal extracellularly and C‐terminal intracellularly, while the C‐terminus of both types of receptors contains phosphorylation and palmitoylation sites, which involve the desensitization process of agonist‐dependent receptors and the formation of the fourth intracellular loop.^11^

## 3 SIGNALING PATHWAYS OF D1RS AND D2RS

In recent years, the intracellular signal transduction pathways of D1Rs and D2Rs have been revealed by reverse transcription‐polymerase chain reaction (RT‐PCR), laser confocal microscopy, patch clamp, gene knockout, and other molecular biological techniques. D1Rs can be coupled to Gs/olf proteins to mediate their physiological functions via the cAMP/PKA/PP‐2A signal transduction pathway.^8^, ^9^, ^10^ D2Rs also regulate intracellular calcium levels by modulating changes in calcium ion activity and thus calcium‐regulated phosphatase (PP2B) protein signaling.^10^, ^12^ Chiefly, the D2L receptor can mediate physiological functions through multiple signaling pathways. After coupling the D2L receptor with Gi protein, it inhibits adenylate cyclase activity, resulting in reduced cAMP production, followed by weakening cAMP‐dependent protein kinase A activation. Then the dephosphorylation of dopamine and cAMP‐regulated phosphoprotein will enhance the activity of protein phosphatase l as well as inhibit cAMP response element‐binding protein and immediate early gene expression, such as fos, jun, and ΔfosB, and further inhibit some effector proteins and ion channel activities of downstream pathways. In addition, the D2L receptor also mediates a macromolecular complex composed of β arrestin2, serine/threonine kinases Akt, and phosphatase PP‐2A.^7^, ^11^, ^13^

## 4 EXPRESSION OF D1RS AND D2RS IN THE CNS

**Figure 1:**
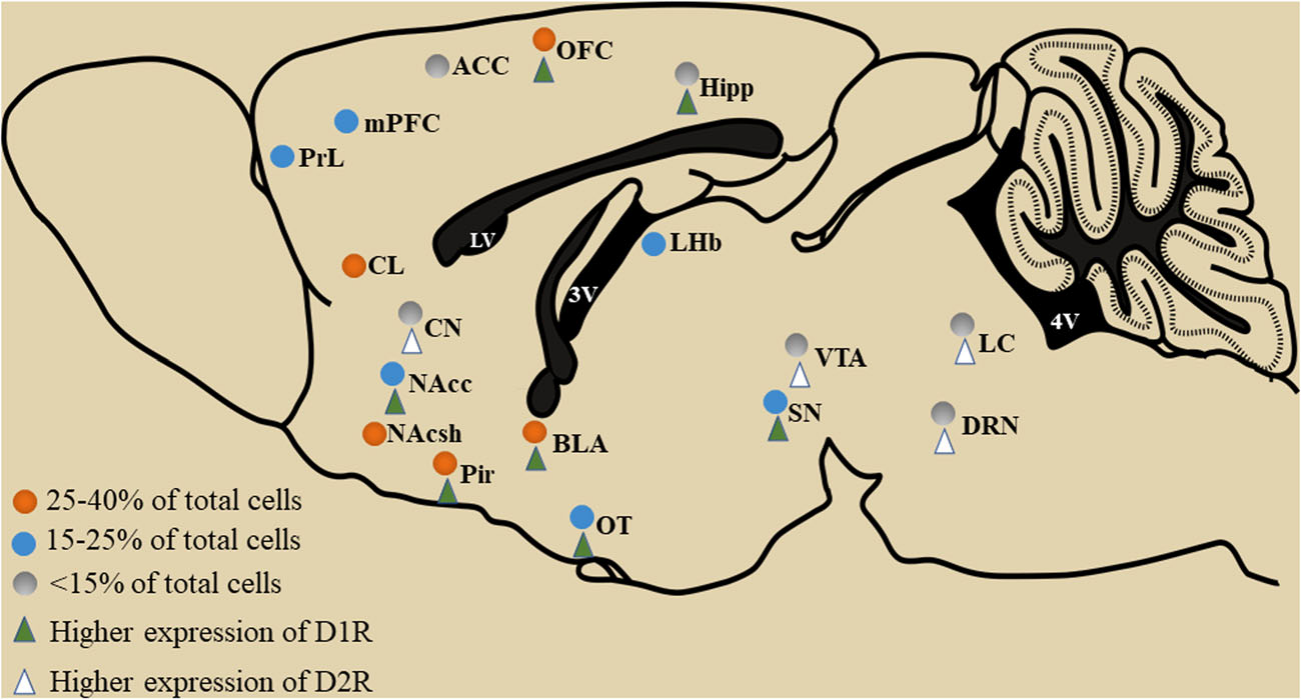
Distribution of dopamine D1Rs and D2Rs in rat brain regions [Color figure can be viewed at wileyonlinelibrary.com]

The distribution of D1Rs and D2Rs is evaluated by in situ proximity ligation assay. D1R and D2R are enriched in nucleus accumbens (NAc), piriform cortex, orbitofrontal cortex, and claustrum, accounting for 25%–40% of the total cells, and then in the substantia nigra, lateral habenula, amygdala, and medial prefrontal cortex (mPFC), and a small amount are expressed in the olfactory tubercle, hippocampus, ventral tegmental area (VTA), caudate nucleus, dorsal raphe nucleus and locus coeruleus (LC).^14^, ^15^ D1Rs and D2Rs are highly expressed by medium spiny neurons (MSNs) of the striatum. Considerable numbers of neurons expressing D1R or D2R are located in different regions of the cortex, as D1Rs are mainly in the deep layers and D2Rs are primarily found in the superficial layers.^16^ It was detected by fluorescence in situ hybridization (FISH) that the expression of D1Rs is higher than that of D2Rs in the piriform cortex, orbitofrontal cortex, insular cortex, nucleus accumbens, amygdala, olfactory tubercle, hippocampus and substantia nigra. A huge amount of neurons expressing D2Rs in the dorsal striatum, LC, and VTA was higher than that of D1Rs. The co‐localization rate of D1Rs and D2Rs in the nucleus accumbens, olfactory tubercle, piriform cortex, and amygdala was approximately 10%–20%. D1Rs and D2Rs were dramatically low in the cingulate cortex, VTA, SN, and LC, with almost no co‐localization^14^ (Figure 1).

The study on the distribution of D1Rs and D2Rs in different regions of CNS lays a foundation for further investigation of the function and role of D1Rs and D2Rs in physiological sleep and general anesthesia.

## 5 THE ROLE OF D1RS AND D2RS IN SLEEP AND WAKEFULNESS

DA receptors have been shown to mediate sleep and wakefulness in a number of studies.^15^, ^17^, ^18^, ^19^ For instance, a study showed that D1R agonist SKF38393 effectively reduced excessive daytime sleepiness and restored rapid eye movement sleep to baseline in monkeys with Parkinson's disease.^20^ By using a highly automated sleep‐wake behavior bioanalytical system, it is observed that D1Rs and D2Rs antagonists blocked the arousal effect of low‐dose DA neuron agonists modafinil (22.5 mg/kg or 45 mg/kg). Interestingly, when given high‐dose modafinil (90 mg/kg or 180 mg/kg), D2R antagonists significantly attenuated the effect of arousal by using modafinil, but D1R antagonists did not affect.^17^ These results suggest that D1Rs and D2Rs are indispensable for the arousal effect of modafinil. Systemic administration of SKF 38393, also induces natural arousal, which causes a dramatic increase in wakefulness and a reduction of sleep time. These results suggest that D1Rs and D2Rs are vital for the arousal process.

During the light phase of the light‐dark cycle, the rapid eye movement sleep (REMs) was significantly reduced when the dopaminergic agonists SKF38393, bromocriptine or quinpirole was microinjected into the dorsal raphe nucleus (DRN). Additionally, bromocriptine and quinpirole remarkably augment wakefulness. In contrast, local administration of SCH23390 and schapride, the D1R and D2R antagonist, into DRN remarkably increased REMs and REM cycles. SCH23390 and sulpiride pretreatment eliminated the effects of SKF38393 and bromocriptine on sleep variables, respectively. Collectively, dopaminergic neurons of VTA and SNc participate in the regulation of wakefulness and REMs by DRN serotoninergic neurons.^15^

In another study, activate D1R‐expressing neurons in the NAc (NAc^D1R^) by optogenetic induced immediate transition from non‐rapid eye movement sleep (NREMs) to wakefulness, whereas chemogenetic activation of NAc^D1R^ neurons generates wakefulness and silencing of NAc^D1R^ neurons suppresses arousal; chemogenetic inhibition of NAc^D2R^ neurons in mice, arousal increased and NREM and REM sleep decreased significantly, while activation of NAc^D2R^ neurons by the same method resulted in the opposite effect, which suggests that the dopaminergic system may regulate sleep‐wake via dopamine receptors in NAc. Collectively, NAc^D1R^ neurons promote arousal and NAc^D2R^ neurons promote sleep.^18^

Interestingly, a D2R agonist (apomorphine, bromocriptine, quinpirole) exert a biphasic effect, with low doses reducing wake and augmenting slow‐wave sleep (SWS) and REMs, whereas high doses produced opposite effects.^15^ In another study, a genetically engineered method was used to explore the effects of D2R during natural sleep and wakefulness. The arousal state of D2R knockout mice was interrupted by recurrent SWS, resulting in difficulty in maintaining arousal, compared with the contrast group. Administration of dopamine transporter antagonists to D2 receptor knockout mice significantly attenuated their wake‐promoting effects.^21^, ^22^

Chemogenetic activation of VTA dopaminergic neurons facilitates wakefulness, increases the duration of arousal, and reduces the number of awakening attacks. Preprocessing with raclopride, the antagonist of dopamine D2/D3 receptors, completely abolished the increased arousal caused by VTA activation, whereas the D1 receptor antagonist SCH23390 did not. It suggests that activating the VTA dopamine neurons can promote wakefulness via D2/D3 receptors.^19^

Therefore, it can be speculated from these studies that D1Rs have dose‐dependent awakening effects, while D2Rs exhibit biphasic effects.

## 6 THE ROLE OF D1RS AND D2RS IN GENERAL ANESTHESIA

General anesthesia‐induced unconsciousness is highly similar to the process of natural sleep‐induced unconsciousness. Electroencephalography (EEG) studies show that anesthetic‐induced unconsciousness is a rapid transformation from the brain waking to sleeping state^23^, ^24^; the brain experiences γ waves, δ waves, and θ waves^25^, ^26^, ^27^ in sleep and anesthesia states. Brain activity also alternates between the fast‐wave sleep and slow‐wave sleep phases of natural sleep during anesthetic‐induced unconsciousness.^28^, ^29^ Brain nuclei involved in the anesthetic‐induced unconsciousness were involved in the natural sleep process as well in a brain functional imaging study.^30^ Therefore, D1Rs and D2Rs may indirectly regulate consciousness during general anesthesia through sleep‐wake nuclei or pathways.

A whole range of studies has indicated that DA receptors play a crucial part in general anesthesia.^4^, ^5^, ^31^, ^32^ Current research suggests that Ritalin, a DA and norepinephrine reuptake inhibitor, can facilitate the recovery from isoflurane or propofol anesthesia by activating the dopaminergic pro‐arousal pathway.^33^ By using behavioral and EEG methods, Boly and colleagues found that Ritalin could reduce the recovery time of rats after single propofol infusion, promoting the behavioral arousal in rats under continuous propofol infusion, and the EEG changed from δ wave (<4 Hz) to θ wave (4~8 Hz) and β wave (12~30 Hz).^33^ This effect could be inhibited by the DA receptor inhibitor droperidol. In rats, microinjection of Ritalin decreases the emergence from isoflurane anesthesia and promotes cortical arousal.^34^ Although the specific mechanism of Ritalin promoting the recovery from general anesthesia is still unclear, it is preliminarily speculated that during isoflurane and propofol anesthesia, Ritalin may induce emergence by acting on dopamine and its receptors at the neural circuit level rather than molecular level antagonism, since propofol and isoflurane are two general anesthetics with different molecular mechanisms.

D2S receptor is highly expressed in the soma and dendrites of VTA and plays a negative feedback inhibitory role to regulate DA synthesis and release, which may be related to emergence from general anesthesia. Electrical stimulation of VTA can induce recovery from isoflurane and propofol general anesthesia. During isoflurane and propofol anesthesia, Ritalin administration and electrical stimulation of VTA can reduce the power of δ band and increase the power of θ band in cortex EEG in rats, and increase the power of β band during propofol anesthesia.^35^ However, Ritalin can increase the power of β band during isoflurane anesthesia, but electrical stimulation of VTA does not. It is generally believed that electrical stimulation of the VTA region activates the neurons near the electrode, and the dopaminergic neurons in VTA release DA neurotransmitters after excitation, which act on the D1 Rs and D2Rs in the CNS, then finally lead to the recovery from general anesthesia.

Randomly microinjecting D1R agonist (chloro‐APB), D1R antagonist (SCH23390), and saline into nucleus basalis(NB) during the period of induction and recovery time of propofol anesthesia demonstrated that microinjection of chloro‐APB accelerated the recovery from propofol anesthesia without affecting the induction of anesthesia, and simultaneously decreased the ratio of δ and increased α and β ratios in the prefrontal cortex in EEG. While SCH23390 produced opposite effects, increased δ ratio, and decreased β ratio. Microinjecting the saline in NB did not influence the process of induction or recovery of propofol anesthesia. The studies above indicated that D1Rs in NB are involved in modulating the emergence from propofol anesthesia, but not affecting the induction period.^5^

Previous studies have indicated that electrical stimulation of VTA, the major dopaminergic nuclei, resulted in membrane depolarization of putative pyramidal cells in the rat's mPFCunder urethane anesthesia.^36^ What's more, electrical stimulation of VTA continuously eliminates slow‐wave activity (SWA) and induces low amplitude rapid (LAF) network rhythm, similar to REMs. Systemic infusion of D1‐like receptors antagonist inhibited the effects of VTA stimulation in all mPFC subregions, implying that DA mediates the shift from SWA to REM‐like state induced by VTA stimulation through D1‐like receptors.^36^

In another study, microinjecting D1R agonist or antagonist facilitated or delayed the awakening from isoflurane anesthesia in young mice by activating or inhibiting D1Rs in the NAc shell, respectively. Nonetheless, this modulation capacity of D1Rs in the NAc shell declined in aged mice.^37^ Meanwhile, downregulation of D1R expression in the NAc shell was detected in the aged brain, which was associated with delayed awakening in aged mice under isoflurane anesthesia.^37^ Using whole‐cell patch clamp to further investigate the underlying cellular mechanism of D1Rs on MSNs under propofol anesthesia, the results primarily demonstrated that propofol raise the frequency and prolonged the decay latency of spontaneous inhibitory postsynaptic currents (sIPSCs) and miniature IPSCs (mIPSCs) of D1R‐expressing MSNs (D1‐MSNs).^32^ D1R agonist reversed the effect of propofol on the frequency of sIPSCs and mIPSCs, and which effect have eliminated by pre‐application of D1R antagonist SCH‐23390. Taken together, D1R modulating the activation of NAc MSNs is critical for emergence from propofol‐ or isoflurane‐induced unconsciousness.^32^ Optogenetic and chemogenetic activation of NAc^D1R^ neurons, respectively, the induction period is extended while the recovery time is shortened under sevoflurane anesthesia in mice, while inhibition of these neurons yielded opposite results. The NAc may promote arousal from anesthesia via its expressed dopamine receptors.

Intravenous droperidol, the D2 receptor antagonist, augments both α and δ‐θ bicoherences while shifting the α‐bicoherence peaks to lower frequencies, and enhances the effect of sevoflurane anesthesia on the EEG via γ‐aminobutyric acid‐mediated oscillatory network regulation.^4^

From these studies, D1R and D2R play an important role in the regulation of general anesthesia. D1R mainly promotes awakening, but the role of D2R is controversial and needs to be further clarified.

## 7 CONCLUSION

**Figure 2:**
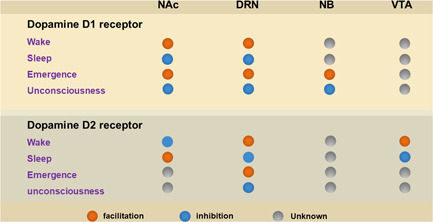
The role of intracerebral dopamine D1Rs and D2Rs in sleep‐wake and general anesthesia‐induced unconsciousness/emergence [Color figure can be viewed at wileyonlinelibrary.com]

To sum up, with the development of immunofluorescence electron microscopy, neurophysiology, optogenetics, and chemogenetics, we have gained more insight into the function of dopamine and its receptors. Among them, D1 and D2 receptors, as two important dopamine receptors in the CNS, are significant for regulating the process of sleep‐wake and accelerating the emergence from general anesthesia (Figure 2). However, the mechanism of consciousness change remains to be further investigated. By revealing the physiological function, pharmacological characteristics and clinical value of D1Rs and D2Rs, we will further understand the mechanisms of D1Rs and D2Rs in promoting recovery from general anesthesia. It is of great significance for us to create novel medications targeting D1Rs and D2Rs, to promote postoperative recovery from general anesthesia and reduce complications, including delayed awakening, delirium, and postoperative cognitive impairment throughout the recovery phase.

## CONFLICT OF INTERESTS

The authors declare that there are no conflict of interests.

## ETHICS STATEMENT

The ethics statement is not available.

## AUTHOR CONTRIBUTIONS

Jie Zhang and Yi Zhang contributed to the main conception, submission, and resource collecting; Jie Zhang, Jia Li, Cheng‐Xi Liu, Huan Gui, and Cheng‐Dong Yuan contributed to drafting and editing; Yi Zhang finalized the review and approved the final version.

## TRANSPARENCY STATEMENT

The authors affirm that this manuscript is an honest, accurate, and transparent account of the study being reported; that no important aspects of the study have been omitted; and that any discrepancies from the study as planned (and, if relevant, registered) have been explained.

## Data Availability

Data sharing not applicable to this article as no datasets were generated or analysed during the current study.
